# External reviews: making an eye service safer

**Published:** 2019-09-10

**Authors:** Beth Barnes, Bernie Chang, Melanie Hingorani

**Affiliations:** 1 Head of Professional Support: Royal College of Ophthalmologists, London, UK.; 2 Chair of External Service Reviews: Royal College of Ophthalmologists, London, UK.; 3 Chair of Professional Standards: Royal College of Ophthalmologists, London, UK.

**Over-stretched health services can struggle to ensure that patient safety and quality of care is of a consistently high standard, which sometimes results in harm to a patient. An external review can provide helpful guidance for improvement.**

**Figure:**
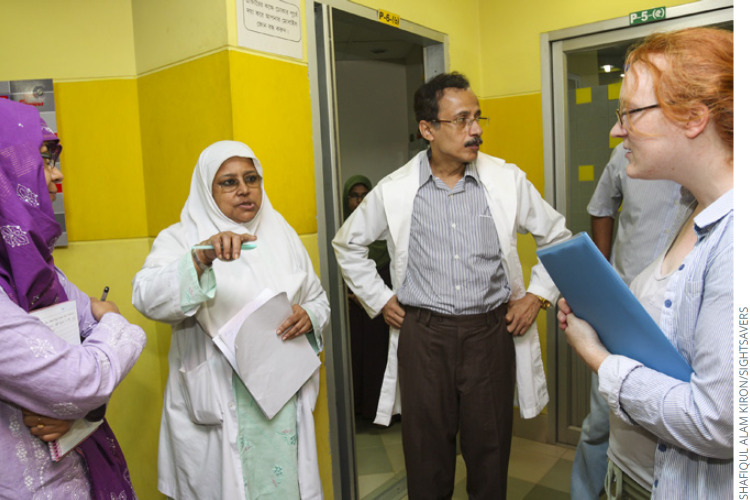
It is vital to involve staff members when planning a service review. BANGLADESH

When something goes wrong, it is important to admit it and be transparent. Patient complaints, concerns about standards of care, adverse outcomes, medical errors and ‘never’ events must be taken seriously and lessons must be learnt from them.

A ‘never’ event^1^ is defined in the UK as an adverse event in medical care that is identifiable, preventable and can have serious consequences for patients. Those relevant to ophthalmology are:

- Wrong site/side surgery (includes wrong patient)
- Wrong implant surgery
- Retained foreign object after surgery.

A no-blame culture and a ‘whistleblowing’ policy (which states that individuals are able to raise concerns without fear of prejudice) is important for transparency. Medical personnel also have a duty of candour (p. 21) which means they are responsible for reporting incidents.

Although it is common for organisations to be aware of risks to patient care and safety and to make attempts to address these, this can be difficult amidst the day-to-day pressures and challenges of a busy hospital or eye unit. Lack of time to make all the changes needed, or a lack of independent and objective input, can result in further under-performance and a negative impact on patients, which causes stress and difficulties for staff members. An independent and external service review can help.

## Do we need an external review?

External reviews are often triggered due to concerns about patient safety. This might come from a team's own reflection of how it is performing, from an inspection that has highlighted areas of improvement, or – unfortunately – when a medical error or incident has occurred.

Doing nothing is not a good option. A continuing series of errors will lower morale, damage the reputation of the service, and may lead to court cases.

## How does an external review work?

An external review involves a team of independent and objective experts visiting the hospital or eye unit to look at:

- Current performance
- The way the service is run
- The challenges faced, e.g., finances, human resources, or issues with higher management
- What needs to change in order to improve patient outcomes and safety.

The review is carried out in close collaboration with the ophthalmology team being reviewed. The external review team will highlight areas of concern and make practical recommendations for improvement; all of which is written in a report that is confidential to the hospital. If serious issues are identified and patients are possibly coming to harm, the review group may have to act accordingly and report this to relevant authorities.

Above all, the central ethos of an external review is one of shared learning to improve the outcomes for patients.

## Inviting a review

Inviting an external review can seem intimidating. However, external reviews are collaborative and supportive in nature, and the report is usually confidential to the eye unit/hospital. The unit requesting the review should have the opportunity to agree who the reviewers are, and should be involved in every aspect of the process. The terms of reference for the review must also be agreed by all parties. If there is no external review service in your country or region, it may be worthwhile contacting your department of health, or national professional body or association, to suggest they set up an external review service.

## Testimonials from UK hospitals

“The review report highlighted shortcomings within the service and other safety issues. All recommendations taken on board and implemented. No safety or service related problems have since been identified. A well run system is in place with high level of patient satisfaction. Extremely valuable for safe and efficiently running service meeting all national standards for care.”

“The review has been a very useful exercise in terms of getting an external opinion on the service. It has instigated discussion within the team and enabled us to formulate our own action plan to take forward the recommendations. This has coincided with a change in the management team, which has had a positive impact, in terms of enabling change.”

“On behalf of the department, we found the review extremely helpful […] it highlighted many areas that required increased input with a big emphasis on the development of allied health professional roles; something we've taken on board […] finally, we're beginning to see action at a senior level on this, with engagement that we've just not seen before.”

## How to provide a review service

For a review to be credible and realistic, a review service must retain its independence, objectivity and impartiality, and it must be open and transparent in all its dealings.^2^

It is important to develop a framework that sets out how the service will be governed and what processes will be followed. Aspects to consider include data security, training for reviewers, how to generate useful reports, and fees. A review may take anything from two days to more than two weeks, so consider the resources and expenses needed and plan for the costs accordingly.

### Who should be on the review team?

It is a privilege and a responsibility to be a member of a review team. In the UK, feedback from reviewers suggest that the reviews are a valuable learning experience for them and for the organisations requesting assistance.

It is important that members of the external review team have extensive experience and are generally well-respected professionals in their field. However, training is required and is important to ensure that members understand how to conduct interviews, assess problems and make recommendations.

The team can include doctors, lay reviewers and any other allied health professionals (usually a team of three). It is important to ensure that there are no conflicts of interest, so the review can be carried out without bias. It is very important that the unit requesting the review has an opportunity to agree who the reviewers are.

### Top tips

- Follow your governance and process framework.
- Remember that this can be an intimidating and stressful time for the unit, so be open and friendly and reassure them that you are there to help and not to judge.
- Before the day of the visit, discuss the issues and requirements of the review with organisation leaders. This includes developing the specific terms of reference and planning how the review team will approach the review.
- Undertake the review in a timely manner; this will minimise stress and expense for the organisation, the ophthalmology team, and the reviewers.
- Agree the roles of each member of the review team, e.g., who will take the notes, and agree the possible questions to ask interviewees.
- Information and evidence should be gathered from public sources and from the organisation throughout the review, and this should be analysed before, during and after the visit. Don't be afraid to carry on asking questions until you have enough information.
- Use a standard template for the report.
- Give the unit, and the individuals interviewed, every opportunity to fully engage with the process, to understand the reason for the review and to consent to it. Encourage individuals to present their views in interviews. They should be told what other evidence has been provided to the review team.
- Check your findings and recommendations to ensure they are robust and do not include *unconscious bias* (see Further reading).
- Allow an opportunity for the organisation being reviewed to correct any factual errors, but not allow them to influence the report itself; this must remain independent.
- The reports and analysis should add value with clear judgements and, where appropriate, recommendations.
- The confidentiality of individuals providing information should be respected, but the over-riding aim is to assess and put in place recommendations to address identified patient safety issues.
- At the end of the visit, provide an overview and preliminary recommendations to the team, in person – including quick wins and immediate actions to take. Then follow up with the full report.

**Figure:**
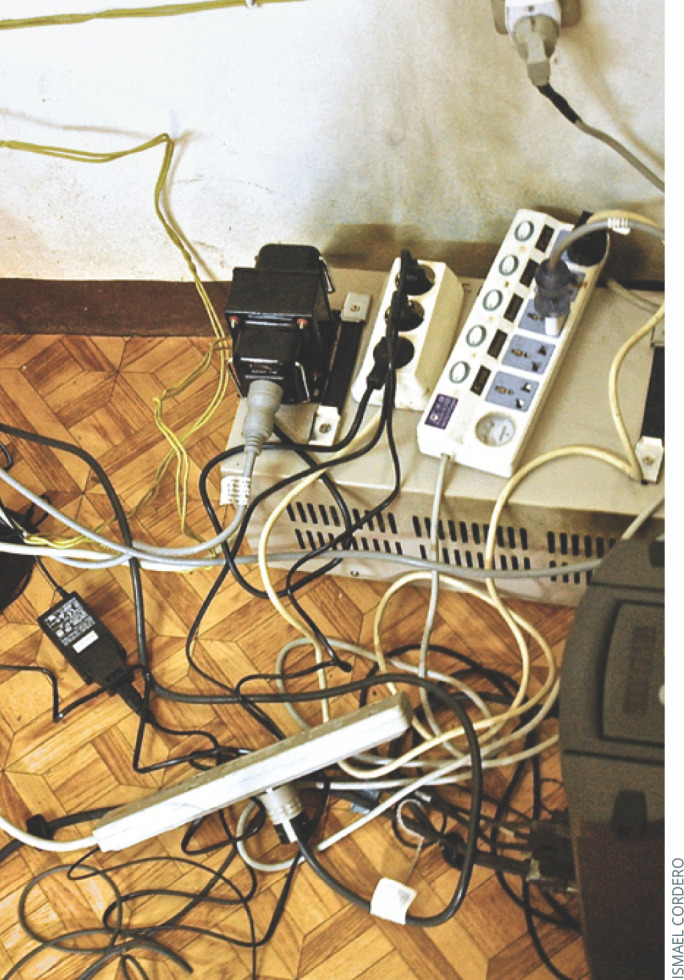
Dangerous equipment has been identified in some external reviews.

**The RCOphth review service**

Over the last 12 years, the RCOphth has assisted over 40 organisations with formal review services and provided informal advice, assessments and recommendations to many more. The overriding aims of each review are to ensure the highest standards of care and patient safety are recognised and being delivered.

In most reviews, the RCOphth external service review team was often simply confirming the issues that staff had already recognised and, in some cases, already addressing. Often, it was clear that ophthalmology department staff were committed to high standards of patient care and welcomed the support and recommendations we provided. Our experience in performing such reviews has shown that there are often common or recurring issues. The RCOphth shares these identified themes for learning and improvement^3^ more widely, so that other units can benefit.

The most rewarded part of the process usually comes sometime after the review report has been submitted. We request follow-up information after six months and about 50% of organisations have responded to these requests. Ongoing engagement is improving, and we also provide ongoing support if required.
